# Comparison of efficacy between transforaminal and interlaminar endoscopic discectomy for single-level lumbar disc herniation

**DOI:** 10.1097/MD.0000000000046711

**Published:** 2025-12-26

**Authors:** Yu Wang, Jingran Guo, Shan Li, Ye Yuan, Jing Zhao

**Affiliations:** a Spine Surgery Department 1, The Second Hospital of Tangshan, Tangshan, China; b Section of Inner Infection Prevention, The Second Hospital of Tangshan, Tangshan, China; c Department of Pathology, College of Basic Medicine, North China University of Science and Technology, Tangshan, China.

**Keywords:** Interlaminar endoscopic lumbar discectomy, lumbar disc herniation, Oswestry disability index, transforaminal endoscopic lumbar discectomy, visual analogue scale

## Abstract

This retrospective study compared the efficacy of transforaminal endoscopic lumbar discectomy (TELD) and interlaminar endoscopic lumbar discectomy (IELD) for single-level L4–L5 lumbar disc herniation. A total of 208 patients were included, with 101 undergoing TELD and 107 undergoing IELD between August 2022 and August 2024. Baseline demographic and clinical characteristics were comparable between groups. Perioperative analysis showed that TELD required a longer operative time and more intraoperative fluoroscopic exposures than IELD, whereas hospital stay and complication rates were similar. At 12-month follow-up, both techniques achieved significant improvements in visual analogue scale and Oswestry disability index scores relative to baseline. However, IELD yielded lower residual pain and better functional recovery, reflected by significantly lower final visual analogue scale and Oswestry disability index scores compared with TELD. These findings suggest that both procedures are safe and effective for L4–L5 lumbar disc herniation, but IELD may provide greater surgical efficiency and superior long-term functional outcomes, emphasizing the importance of anatomy-guided approach selection.

## 1. Introduction

Lumbar disc herniation (LDH) is among the most common causes of lower back pain and radiculopathy, affecting a substantial proportion of the working-age population worldwide. The L4–L5 intervertebral level is particularly susceptible due to its anatomical position and the significant mechanical stress imposed during daily activities.^[1]^ Symptomatic LDH not only impairs physical function but also contributes to substantial socioeconomic burdens, including loss of productivity and increased healthcare utilization. Although conservative treatment modalities such as physical therapy, pharmacological management, and epidural steroid injections remain first-line approaches, a subset of patients with persistent or progressive neurological symptoms requires surgical intervention.^[2,3]^

Traditional open discectomy has long been considered the standard surgical option. However, the associated muscle dissection, blood loss, and postoperative morbidity have stimulated the development of less invasive alternatives. In this context, spinal endoscopic techniques have gained prominence as they minimize tissue trauma while preserving spinal stability. Endoscopic discectomy allows direct visualization and targeted removal of herniated disc material through a minimal access corridor, thereby reducing recovery time and postoperative complications. Two widely employed approaches in endoscopic spine surgery are the transforaminal endoscopic lumbar discectomy (TELD) and the interlaminar endoscopic lumbar discectomy (IELD).^[4,5]^ Both approaches have demonstrated favorable clinical outcomes, yet their relative efficacy remains a matter of ongoing investigation. The transforaminal approach provides access to the herniated disc through the intervertebral foramen, enabling removal of disc fragments without significant disruption of posterior spinal structures. It is particularly advantageous for foraminal and extraforaminal herniations. Nevertheless, TELD may be technically challenging at certain levels, particularly the L5–S1 level, due to the high iliac crest or narrow foraminal space. Conversely, the interlaminar approach utilizes the natural anatomical window between adjacent laminae, offering a more direct trajectory to central and paracentral herniations.^[6–8]^ The L4–L5 level is especially suited for IELD because of its relatively wide interlaminar space. While both techniques are minimally invasive, their indications, learning curves, complication profiles, and long-term functional outcomes may differ considerably.

Given the clinical importance of optimizing surgical strategies for LDH, particularly at the L4–L5 level where herniations are most prevalent, a direct comparative evaluation of TELD and IELD is warranted. Clarifying the relative efficacy of these approaches in terms of pain reduction, functional improvement, perioperative parameters, and complication rates may provide critical guidance for surgical decision-making and patient counseling. Therefore, the present study aims to compare the efficacy and safety of transforaminal versus interlaminar endoscopic discectomy for single-level L4–L5 lumbar disc herniation.

## 2. Methods

### 2.1. Study design

This study was approved by the Ethics Committee of The second hospital of Tangshan.This retrospective study included patients diagnosed with single-level LDH at the L4–L5 segment who underwent surgical treatment at our institution between August 2022 and August 2024. Eligible patients were aged 18 to 70 years, had radiological confirmation of L4–L5 disc herniation by magnetic resonance imaging (MRI) and/or computed tomography, and presented with corresponding clinical symptoms, including low back pain and/or unilateral lower extremity radicular pain, with or without neurological deficits. All patients had failed standardized conservative management, such as physical therapy, nonsteroidal anti-inflammatory drugs, or epidural steroid injections, for a minimum of 6 to 8 weeks, and demonstrated an indication for surgery due to persistent pain, progressive motor weakness, or significant impairment of quality of life. Patients were excluded if they exhibited multilevel or recurrent disc herniation, lumbar spinal stenosis, spondylolisthesis, spinal deformity, segmental instability, or other degenerative spinal conditions requiring additional procedures. Additional exclusion criteria included prior lumbar spine surgery at any level, severe calcification or ossification of the herniated disc precluding endoscopic removal, or the presence of spinal infections, tumors, fractures, or inflammatory diseases. A total of 208 patients met the eligibility criteria and were enrolled in the study. Based on the surgical approach, patients were stratified into the TELD group (n = 101) and the IELD group (n = 107). The study was conducted in accordance with the ethical standards of the Declaration of Helsinki and was approved by the institutional medical ethics committee. Written informed consent was obtained from all participants prior to surgery.

### 2.2. Treatment protocol

TELD: Patients in the TELD group underwent the procedure under local anesthesia. With the patient placed in the prone position and arranged in the “jackknife” posture, anatomical landmarks including the iliac crest line, spinous process midline, and target intervertebral space were identified and marked. After routine skin preparation and draping, a spinal needle was inserted 8 to 10 cm lateral to the midline at an angle of 5° to 10° to the horizontal plane of the intervertebral space. The needle was advanced through the skin, subcutaneous tissue, deep fascia, and musculature until reaching the ventral aspect of the L5 articular process. Under C-arm fluoroscopic guidance, the needle position was confirmed, and a guidewire was inserted. Following removal of the needle, a skin incision approximately 0.8 cm in length was made at the guidewire entry site. Sequential soft tissue dilators were introduced, and foraminoplasty was performed when necessary. A working cannula was then inserted and connected to the endoscopic system. The dorsal portion of the ligamentum flavum was carefully removed to expose the nerve root, and decompression was carried out by excising the herniated nucleus pulposus using pituitary forceps. Adequacy of decompression was confirmed by visualization of restored mobility and relaxation of the affected nerve root. Hemostasis was achieved with radiofrequency coagulation, after which the endoscope was withdrawn and the incision closed.

IELD: Patients in the IELD group underwent the procedure under general anesthesia. After being positioned prone in the jackknife posture, routine disinfection and draping were performed. A skin incision of approximately 1.0 cm was made at the intersection of the medial borders of the adjacent pedicles and the horizontal plane of the intervertebral space on the surgical side. Following incision of the skin and subcutaneous fascia, sequential dilators were introduced and a visualized trephine working sleeve was placed with its opening directed toward the superior lamina. Under endoscopic visualization, local soft tissues were cleared to expose the superior and inferior laminae as well as the ligamentum flavum. Using a visualized trephine in a counterclockwise direction, a “U-shaped” laminotomy was created to expose the cranial and caudal insertions and lateral border of the ligamentum flavum. The medial margin of the superior articular process was resected with rongeurs to enlarge the laminotomy window. The nerve root was then mobilized medially using a microdissector after careful dissection of adhesions along its lateral margin. The working cannula was advanced into the spinal canal lateral to the nerve root, allowing access to the annular rupture site. The herniated disc material was completely excised under direct visualization by sequential adjustment of the working cannula in superior, inferior, medial, and lateral directions. Adequate decompression was confirmed when the nerve root regained normal mobility and tension. Hemostasis was performed as required, and the incision was closed.

Postoperative management: At 12 hours postoperatively, patients were permitted to ambulate while wearing a rigid lumbar brace. Hospital discharge typically occurred within 1 to 2 days after surgery. Normal daily activities and occupational tasks were gradually resumed at approximately 6 weeks postoperatively. All patients were followed up for a minimum of 12 months to monitor clinical recovery and potential recurrence.

### 2.3. Data collection

Perioperative data were systematically collected for all patients, including operative time, defined as the duration from the initial skin incision to wound closure; the number of intraoperative fluoroscopic exposures, reflecting both surgical accuracy and radiation exposure; postoperative length of hospital stay, recorded as the number of days from surgery to discharge; and the occurrence of surgical complications, which were carefully documented and categorized according to type and severity, such as dural tears, nerve root injury, excessive bleeding, wound infection, or recurrence identified during hospitalization.

Pain intensity was evaluated using the visual analogue scale (VAS), a 10-point numerical rating system ranging from 0 (no pain) to 10 (worst imaginable pain). Both low back pain and radicular leg pain were assessed preoperatively and again at the final follow-up (12 months postoperatively). The degree of improvement in VAS scores was employed to quantify the efficacy of surgical intervention in alleviating pain symptoms.

Lumbar function was assessed with the Oswestry disability index (ODI), a validated questionnaire designed to evaluate disability related to lumbar disorders. The ODI comprises 10 domains, including pain intensity, personal care, lifting, walking, sitting, standing, sleeping, social life, traveling, and employment or homemaking. Each item is scored from 0 to 5, with higher scores indicating greater disability. The total ODI score was converted into a percentage according to the formula: ODI (%) = (total score/maximum possible score) × 100. Evaluations were conducted preoperatively and at the 12-month follow-up, and changes in ODI values were used to determine functional recovery following surgery.

All patients were followed through standardized outpatient visits and telephone interviews, with assessments scheduled at baseline and at the 12-month postoperative time point. Clinical data were collected by spine surgeons who were blinded to the surgical approach in order to minimize assessment bias and ensure the reliability of outcome measurements.

### 2.4. Statistical analysis

All statistical analyses were performed using SPSS software (version 28.0; IBM Corp., Armonk, NY). Continuous variables, including operative time, number of intraoperative fluoroscopic exposures, length of hospital stay, VAS scores, and ODI values, were first tested for normality using the Shapiro–Wilk test. Data with a normal distribution were expressed as mean ± standard deviation and compared between groups using the independent-samples *t* test, whereas non-normally distributed data were presented as median with interquartile range and analyzed with the Mann–Whitney *U* test. Categorical variables, such as the incidence of surgical complications, were presented as frequencies and percentages, and comparisons between groups were conducted using the chi-square test or Fisher exact test when appropriate. Within-group comparisons of preoperative and postoperative VAS and ODI scores were evaluated using the paired-samples t test or Wilcoxon signed-rank test, depending on the distribution of data. Cases with incomplete data were excluded from the corresponding analyses, and the extent of missing data was minimal (<5% for clinical variables). A 2-tailed *P* value of <.05 was considered to indicate statistical significance.

## 3. Results

### 3.1. Baseline characteristics

A total of 208 patients were enrolled in this study, including 101 in the TELD group and 107 in the IELD group. As shown in Table 1, there were no statistically significant differences in demographic or clinical baseline parameters between the 2 groups (*P* > .05). The distribution of sex and age was comparable, with a similar mean age of approximately 43 years. The duration of symptoms prior to surgery did not differ significantly, and the laterality of disc herniation (left vs right) was evenly distributed across both groups. In terms of general health status, body mass index was nearly identical between groups, and lifestyle-related factors such as smoking history showed no significant variation. Likewise, the prevalence of common comorbidities, including hypertension and diabetes mellitus, was balanced between the 2 cohorts (Table 1).

**Table 1:** Baseline characteristics of patients in the TELD and IELD groups.

Variable	TELD group (n = 101)	IELD group (n = 107)	*t*/*χ*^2^ value	*P* value
Sex (male/female)	55 (54.5%)/46 (45.5%)	58 (54.2%)/49 (45.8%)	0.001	.971
Age (yr)	42.95 ± 8.12	43.12 ± 8.35	0.149	.882
Symptom duration (mo)	6.85 ± 3.08	7.15 ± 3.58	0.646	.519
Side of herniation (left/right)	48 (47.5%)/ 53 (52.5%)	51 (47.7%)/ 56 (52.3%)	0.001	.984
BMI (kg/m^2^)	24.10 ± 2.80	24.40 ± 2.60	0.801	.424
Smoking history, n (%)	28 (27.7%)	32 (29.9%)	0.121	.728
Hypertension, n (%)	15 (14.9%)	17 (15.9%)	0.043	.836
Diabetes mellitus, n (%)	11 (10.9%)	12 (11.2%)	0.006	.941

BMI = body mass index, IELD = interlaminar endoscopic lumbar discectomy, SD = standard deviation, TELD = transforaminal endoscopic lumbar discectomy.

### 3.2. Perioperative outcomes

Perioperative outcomes for the 2 groups are summarized in Table 2. Operative time was significantly longer in the TELD group compared with the IELD group (74.36 ± 14.21 minutes vs 51.62 ± 15.84 minutes, *P* < .001). Similarly, the number of intraoperative fluoroscopic exposures was markedly higher in the TELD group (10.51 ± 5.64) than in the IELD group (3.15 ± 1.58, *P* < .001). These findings indicate that the transforaminal approach required more time and greater reliance on intraoperative imaging than the interlaminar approach. In contrast, the length of postoperative hospital stay was comparable between groups, with both cohorts discharged within approximately 2 days after surgery, and no statistically significant difference was observed (*P* = .794). The overall complication rate was low in both groups, with 9.90% in the TELD group and 9.35% in the IELD group, and no significant difference was found (*P* = .892) (Table 2).

**Table 2:** Comparison of perioperative outcomes between the TELD group and the IELD group.

Variable	TELD group (n = 101)	IELD group (n = 107)	*t*/*χ*^2^ value	*P* value
Operative time (min)	74.36 ± 14.21	51.62 ± 15.84	10.880	<.001
Fluoroscopy times	10.51 ± 5.64	3.15 ± 1.58	12.973	<.001
Postoperative hospital stay (d)	1.75 ± 0.59	1.73 ± 0.51	0.262	.794
Complications, n (%)	10 (9.90)	10 (9.35)	0.018	.892

IELD = interlaminar endoscopic lumbar discectomy, SD = standard deviation, TELD = transforaminal endoscopic lumbar discectomy.

### 3.3. Functional outcomes

Functional recovery, as assessed by VAS and ODI scores, is presented in Table 3. Preoperatively, no significant differences were observed between the 2 groups in either VAS or ODI scores, indicating that baseline pain intensity and disability levels were comparable (*P* > .05). At the final follow-up, both groups demonstrated marked improvement compared with their preoperative status, reflecting the effectiveness of endoscopic discectomy in alleviating symptoms and restoring function. However, significant intergroup differences were noted. Patients in the IELD group reported lower residual pain scores, with a mean VAS of 1.45 ± 0.57 compared with 1.90 ± 0.69 in the TELD group (*P* < .001). Similarly, functional recovery as measured by the ODI showed greater improvement in the IELD group, where the mean score decreased to 10.68 ± 4.05%, compared with 17.40 ± 3.48% in the TELD group (*P* < .001) (Table 3).

**Table 3:** Comparison of VAS and ODI scores between the TELD group and the IELD group.

Variable	TELD group (n = 101)	IELD group (n = 107)	*t* value	*P* value
Preoperative VAS	6.30 ± 1.71	6.27 ± 2.91	0.090	.928
VAS final FU	1.90 ± 0.69	1.45 ± 0.57	5.140	<.001
Preoperative ODI (%)	67.35 ± 5.11	68.15 ± 6.04	1.028	.305
ODI final FU (%)	17.40 ± 3.48	10.68 ± 4.05	12.801	<.001

FU = follow-up, IELD = interlaminar endoscopic lumbar discectomy, ODI = Oswestry disability index, SD = standard deviation, TELD = transforaminal endoscopic lumbar discectomy, VAS = visual analogue scale.

## 4. Discussion

This retrospective comparison of TELD and IELD for single-level L4–L5 LDH demonstrates that both approaches yielded substantial pain relief and functional recovery at 12-month follow-up, with similarly low rates of perioperative complications and equivalent postoperative length of stay. These findings were observed against a balanced baseline—age, sex, symptom duration, laterality, body mass index, smoking status, and cardiometabolic comorbidities did not differ between cohorts—thereby supporting the internal validity of the comparative analyses. Within this context, 2 signals emerged. First, IELD was associated with superior patient-reported outcomes at the final assessment, as reflected by lower residual VAS and ODI scores. Second, TELD entailed longer operative time and substantially more intraoperative fluoroscopic exposures. Together, these results suggest that for L4–L5 pathology the interlaminar route may achieve more effective decompression with greater intraoperative efficiency, whereas the transforaminal route, although safe, is more workflow- and imaging-intensive.

The observed differences in perioperative metrics are anatomically and technically coherent. TELD proceeds through Kambin triangle along an oblique, lateral-to-medial corridor, often necessitating iterative foraminoplasty and frequent fluoroscopic confirmations to maintain a safe trajectory relative to the exiting and traversing roots. These requirements plausibly lengthen operative time and increase fluoroscopy counts. In contrast, IELD at L4–L5 leverages a relatively capacious interlaminar window; the short, orthogonal path to central/paracentral fragments stabilizes endoscopic orientation, reduces the need for trajectory adjustments, and typically limits the number of radiographic checks. Such access characteristics help explain why, despite similar safety and length of stay, IELD achieved better patient-reported outcomes with fewer intraoperative imaging events.^[9,10]^ Because baseline pain and disability were comparable, the between-group differences at follow-up are more likely attributable to approach-specific decompression efficiency rather than preoperative imbalance. Importantly, the similarity in overall complication rates indicates that when performed by experienced teams using standardized perioperative protocols, both techniques are safe. The low incidence of adverse events suggests that, for L4–L5 LDH, the principal trade-offs are not in safety but in operative mechanics (time, fluoroscopy) and the magnitude of residual symptoms. This distinction is clinically relevant: reducing radiation exposure and streamlining operative time have implications for both staff safety and operating room throughput, while improvements in VAS and ODI translate to tangible benefits in patient function and quality of life.^[11]^

Our results align with and extend recently published evidence comparing interlaminar and transforaminal endoscopic routes. Takebayashi and colleagues analyzed L4/5 LDH managed by full-endoscopic discectomy and reported that the interlaminar approach is favored by preoperative radiological factors for intracanal pathology at this level; they also documented approach-specific differences in operative characteristics while maintaining similar safety, echoing our perioperative and outcome signals.^[3]^ Systematic and focused reviews from the last 3 years similarly conclude that, across LDH populations, IELD and TELD achieve broadly comparable safety profiles, with approach-dependent advantages: IELD tends toward shorter operative time and less fluoroscopy in anatomically favorable levels, whereas TELD is advantageous for foraminal/extraforaminal targets—an evidence-based nuance that is concordant with our L4–L5 findings.^[12,13]^

Beyond level-specific series, contemporary comparative work continues to highlight workflow differences that mirror our observations. Studies and meeting abstracts published in 2024 report that, where the interlaminar window is generous, interlaminar endoscopic discectomy can reduce fluoroscopy reliance and improve operative efficiency without compromising outcomes; conversely, transforaminal discectomy may demand more imaging due to the oblique corridor and the need for precise foraminoplasty.^[14,15]^ Although several meta-analyses have focused on L5–S1 or on calcified herniations, settings in which iliac crest height and facet morphology further differentiate the 2 routes, the overarching conclusions remain consistent: clinical efficacy is comparable when indications are respected, while IELD often shows advantages in operative time and fluoroscopy frequency, and TELD retains value for laterally biased disease.^[16,17]^ The convergence of these external data with the present L4–L5 cohort strengthens the generalizability of our principal message: both approaches are effective and safe, but their efficiency and patient-reported endpoints vary in ways that reflect anatomic corridors and technical demands. Finally, recent narrative updates emphasize that IELD affords direct management of concomitant canal pathology and allows tailored laminotomy under endoscopic visualization—features plausibly linked to deeper decompression in central or paracentral herniations at L4–L5. Our finding of superior VAS/ODI with IELD is compatible with this mechanistic rationale and with reports highlighting the versatility of the interlaminar route in intracanal disease.^[18]^

These data support an anatomy-guided decision algorithm at L4–L5 LDH. When preoperative MRI demonstrates central/paracentral compression within the canal and an adequate interlaminar window, IELD should be considered the primary route owing to its association with lower residual pain/disability and reduced fluoroscopy utilization. TELD remains appropriate for laterally biased pathology and in patients for whom a transforaminal trajectory is advantageous for access or comorbidity management. Because complication rates and length of stay are similar, surgical planning can prioritize decompression quality, radiation stewardship, and operating room efficiency. This study has several strengths. First, rigorous baseline comparability between cohorts enhances internal validity and reduces confounding in the absence of randomization. Second, a single surgical team and standardized perioperative pathways limit heterogeneity in technique and postoperative care, focusing the comparison on the approaches themselves. Third, outcomes combine perioperative metrics with validated patient-reported measures (VAS, ODI) at a uniform 12-month time point, enabling a balanced appraisal of safety, efficiency, and effectiveness.

Limitations must be acknowledged. This retrospective design is inherently prone to selection and information bias. Although baseline variables were well matched, unmeasured factors—such as canal diameter, facet hypertrophy, subtle migration patterns, and surgeon preference—may have influenced approach selection and outcomes. Potential confounders including case distribution among surgeons, operator experience, and anatomical variations (e.g., intervertebral foramen width and iliac crest height) were not quantitatively analyzed and should be controlled in future prospective studies. Postoperative MRI was not routinely obtained; thus, imaging-based endpoints of decompression adequacy were not fully assessed. Future research will incorporate standardized postoperative MRI using a residual compression score (0–3) and dural sac cross-sectional area measurements to validate clinical outcomes. A small proportion of patients had incomplete follow-up data, introducing minor attrition bias, though its impact was likely minimal. The 12-month follow-up offers only mid-term insight and cannot assess long-term durability, reherniation, or instability. Radiation exposure was estimated by fluoroscopy counts rather than dosimetry, warranting further evaluation. Being a single-center study may limit generalizability, as learning-curve effects could differentially influence TELD and IELD outcomes. Finally, imaging phenotypes (e.g., cranio-caudal migration, sequestration, calcification) were not stratified, which may refine indication algorithms in future work.

## 5. Conclusions

Both transforaminal and interlaminar endoscopic discectomy are safe and effective for single-level L4–L5 lumbar disc herniation. However, IELD demonstrated shorter operative time, fewer fluoroscopic exposures, and superior improvements in pain and functional outcomes, whereas TELD provided comparable safety but with greater intraoperative complexity. These findings highlight the importance of anatomy-based surgical selection to optimize patient recovery.

## Author contributions

**Conceptualization:** Yu Wang, Jingran Guo, Shan Li, Jing Zhao.

**Data curation:** Yu Wang, Jingran Guo, Shan Li, Jing Zhao.

**Funding acquisition:** Jing Zhao.

**Formal analysis:** Yu Wang, Shan Li, Ye Yuan, Jing Zhao.

**Investigation:** Yu Wang, Jingran Guo, Shan Li, Ye Yuan, Jing Zhao.

**Writing – review & editing:** Shan Li, Ye Yuan, Jing Zhao.

**Writing – original draft:** Ye Yuan, Jing Zhao.
